# Highly encephalitogenic aquaporin 4-specific T cells and NMO-IgG jointly orchestrate lesion location and tissue damage in the CNS

**DOI:** 10.1007/s00401-015-1501-5

**Published:** 2015-11-03

**Authors:** Bleranda Zeka, Maria Hastermann, Sonja Hochmeister, Nikolaus Kögl, Nathalie Kaufmann, Kathrin Schanda, Simone Mader, Tatsuro Misu, Paulus Rommer, Kazuo Fujihara, Zsolt Illes, Fritz Leutmezer, Douglas Kazutoshi Sato, Ichiro Nakashima, Markus Reindl, Hans Lassmann, Monika Bradl

**Affiliations:** Department for Neuroimmunology, Center for Brain Research, Medical University Vienna, Spitalgasse 4, 1090 Vienna, Austria; Department for Neurology, Medical University Graz, Auenbruggerplatz 22, 8036 Graz, Austria; Clinical Department for Neurology, Medical University of Innsbruck, Anichstrasse 35, 6020 Innsbruck, Austria; Department of Neurology, Tohoku University Graduate School of Medicine, 1-1 Seiryomachi, Aobaku, Sendai, 980-8574 Japan; University Hospital for Neurology, Medical University Vienna, Währinger Gürtel 18-20, 1090 Vienna, Austria; Department of Neurology, University of Southern Denmark, Sdr Boulevard 29, Odense, 5000 Denmark; Department of Neurology, Faculty of Medicine, University of Sao Paulo, Av. Dr. Arnaldo, 455-4th floor (sl 4110), 01246-903 São Paulo, Brazil

**Keywords:** CNS inflammation, Neuromyelitis optica, T cells, Aquaporin 4, ENMO

## Abstract

In neuromyelitis optica (NMO), astrocytes become targets for pathogenic aquaporin 4 (AQP4)-specific antibodies which gain access to the central nervous system (CNS) in the course of inflammatory processes. Since these antibodies belong to a T cell-dependent subgroup of immunoglobulins, and since NMO lesions contain activated CD4^+^ T cells, the question arose whether AQP4-specific T cells might not only provide T cell help for antibody production, but also play an important role in the induction of NMO lesions. We show here that highly pathogenic, AQP4-peptide-specific T cells exist in Lewis rats, which recognize AQP4_268–285_ as their specific antigen and cause severe panencephalitis. These T cells are re-activated behind the blood–brain barrier and deeply infiltrate the CNS parenchyma of the optic nerves, the brain, and the spinal cord, while T cells with other AQP4-peptide specificities are essentially confined to the meninges. Although AQP4_268–285_-specific T cells are found throughout the entire neuraxis, they have NMO-typical “hotspots” for infiltration, i.e. periventricular and periaqueductal regions, hypothalamus, medulla, the dorsal horns of spinal cord, and the optic nerves. Most remarkably, together with NMO-IgG, they initiate large astrocyte-destructive lesions which are located predominantly in spinal cord gray matter. We conclude that the processing of AQP4 by antigen presenting cells in Lewis rats produces a highly encephalitogenic AQP4 epitope (AQP4_268–285_), that T cells specific for this epitope are found in the immune repertoire of normal Lewis rats and can be readily expanded, and that AQP4_268–285_-specific T cells produce NMO-like lesions in the presence of NMO-IgG.

**Electronic supplementary material:**

The online version of this article (doi:10.1007/s00401-015-1501-5) contains supplementary material, which is available to authorized users.

## Introduction

**Fig. 1:**
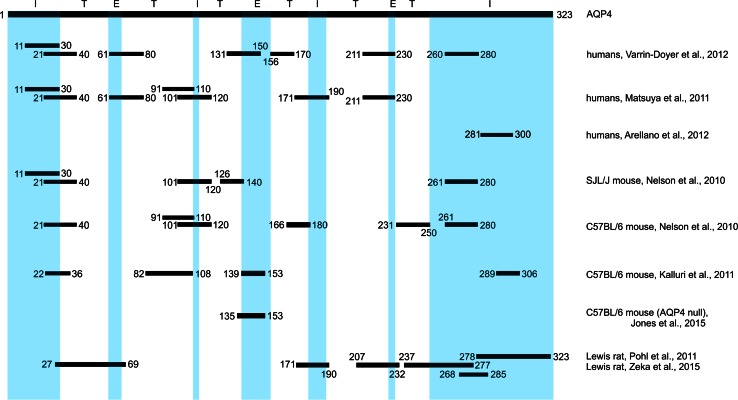
Aquaporin 4 epitopes used for T cell recognition in humans, rats and mice. The *black bar* shown here on the *top* represents the AQP4 isoform M23 and the location of intracellular (I, *blue*), transmembraneous (T, *white*) and extracellular sequences (E, *blue*) in this molecule assigned according to Verkman et al. [39]. The *black*, *numbered lines underneath* show the location and the start/end position of the amino acid sequence of AQP4 epitopes described in humans [4, 23, 38], SJL/J [25] and C57BL/6 [12, 25] mice, C57BL/6 AQP4 null mice [10] and in Lewis rats ( [31] and current publication)

Neuromyelitis optica (NMO) is an inflammatory, astrocytopathic disease of the central nervous system (CNS) [8, 36]. Ever since the hallmark of this disease—the presence of pathogenic autoantibodies in the serum of most NMO patients [19, 20]—has been recognized, a lot of effort has been made to study the role of antibodies and T cells in lesion formation and expansion. Based on these studies we know (1) that in most patients, the pathogenic autoantibodies are directed against aquaporin 4 (AQP4), a water channel enriched on astrocytic endfeet at the perivascular and subpial glia limitans [20, 27], (2) that these antibodies belong to the IgG1 subgroup of immunoglobulins which need T cell help in their formation [20], and (3) that these antibodies have additional requirements for T cells in lesion formation: They need them for opening of the blood–brain barrier to gain access to the CNS parenchyma [6, 7, 14], and they need them to create a CNS environment facilitating antibody-dependent cellular cytotoxicity (ADCC) and complement-mediated cytotoxicity (CDC) against their astrocytic targets [15, 33]. Such an environment is only created by T cells which are activated within the CNS [33], which is in line with the presence of Ox40^+^ CD4^+^ T cells in early lesions of human NMO patients and their experimental counterparts [33]. In order to become activated within the CNS, CD4^+^ T cells must encounter “their” specific, CNS-intrinsic antigen in the context of MHC class II products [13]. Considering the facts that AQP4-specific T cells provide help to the formation of AQP4-specific antibodies, and that AQP4-specific T cells are clonally expanded in NMO patients [23, 38], it is tempting to speculate that the antigen leading to T cell activation within the CNS of NMO patients is AQP4 as well. However, T cell responses against AQP4 target a surprisingly large number of epitopes in humans [22, 37] and even in single patients [23], which is reflected in Lewis rats [32] and in C57BL/6 or SJL/J mice [12, 25] (Fig. 1). Moreover, until recently, only weakly pathogenic AQP4 peptide-specific T lymphocytes could be derived from Lewis rats [32]. These T cells essentially pile up in the meninges, but hardly infiltrate the CNS parenchyma, suggestive of a limited activation of these cells [13, 32, 33]. AQP4-reactive T cells could also be obtained after immunization of C57BL/6 aquaporin-4 null mice with a combination of the human AQP4 extracellular loop peptides AQP4_56–69_, AQP4_135–153_, and AQP4_212–230_ in complete Freund’s adjuvans and subsequent in vitro polarization of the peptide-specific T cells towards a T_H_17 phenotype [10]. These T cells were encephalitogenic, as evidenced by the induction of inflammatory lesions in spinal cords and optic nerves and by the induction of clinical signs of CNS inflammation, but derived from animals which did not have to overcome immunological tolerance, due to the absence of AQP4. In addition, the animals have been challenged with human instead of murine AQP4 [10]. Cumulatively, the findings obtained from both animal models raised the questions whether strongly encephalitogenic AQP4-specific T cells exist at all in the normal immune repertoire, and whether these cells can guide astrocyte-destructive lesions to NMO-typical sites. These questions were addressed in the current study, using Lewis rats as a model organism.

## Materials and methods

### Animals

Lewis rats (7–8 weeks old) were obtained from Charles River Wiga (Sulzfeld, Germany). They were housed in the Decentral Facilities of the Institute for Biomedical Research (Medical University Vienna) under standardized conditions. The experiments were approved by the Ethic Commission of the Medical University Vienna and performed with the license of the Austrian Ministry for Science and Research.

### Characterization of the immunoglobulins used in transfer experiments

The NMO-IgG preparations containing pathogenic AQP4-specific antibodies derived from therapeutic plasmapheresates/sera of two different patients (“NMO-IgG9” and “pt1”; both NMO-IgGs worked equally well). The NMO-IgGs were essentially prepared and purified as described [7], and adjusted to an IgG concentration of 10 mg/ml. The use of it for research was approved by the Ethics Committee of Tohoku University School of Medicine (No. 2007-327) and by the Regional and National Ethical Committee of Hungary (3893.316-12464/KK4/2010 and 42341-2/2013/EKU). The normal human IgG preparation used as a negative control was commercially available (Subcuvia™, Baxter, Vienna), and was also diluted with phosphate-buffered saline (PBS) to an IgG concentration of 10 mg/ml prior to use.

### Antigens

For immunization and T cell isolation/propagation, large peptides or fusion proteins containing predicted epitopes [32] were used (Table 1). These peptides were synthesized by Centic Biotec (Heidelberg, Germany), or, in the case of the human AQP-4 peptide AQP-4_278–323_, were expressed in E. coli using the pBAD/TOPO ThioFusion Expression System (Invitrogen, Carlsbad, CA, USA) and purified as described [32].

**Table 1:** Peptide and epitope sequences

Peptide	Amino acid sequence	Epitopes contained
AQP4_27–69_	KGVWTQAFWKAVTAEFLAMLIFVLLSVGSTINWGGSENPLPVD	p33–41: AFWKAVTAE p55–63: STINWGGSE
AQP4_171–190_	VFTIFASCDSKRTDVTGSVA	p176–184: ASCDSKRTD
AQP4_207–232_	YTGASMNPARSFGPAVIMGNWENHWI	p220–228: PAVIMGNWE
AQP4_237–277_	PIIGAVLAGALYEYVFCPDVELKRRLKEAFSKAAQQTKGSY	p241–249: AVLAGALYE p249–257: EYVFCPDVE
AQP4_268–285_	KAAQQTKGSYMEVEDNRS	p271–279: QQTKGSYME p273–281: TKGSYMEVE
AQP4_278–323_^a^	MEVEDNRSQVETDDLILKPGVVHVIDVDRGEEKKGKDQSGEVLSSV	p297–305: GVVHVIDVD p300–308: HVIDVDRGE

Unless otherwise stated, the peptides/epitopes and amino acid sequences derive from the rat AQP4 sequence (GI:5019990)

^a^This peptide and the corresponding amino acid sequences and epitopes derive from the human AQP4 sequence (GI:4502181)

For specificity tests (see below), also full-length human M23 AQP4 (gene bank accession number: NP-004019) was used, which has 100 % identity to the rat epitopes contained in AQP4_207–232_ (PAVIMGNWE) and AQP4_268–285_ (QQTKGSYME and TKGSYMEVE), and contains the human sequence of AQP4 recognized by AQP4_278–323_-specific T cells (GVVHVIDVD and HVIDVRGE, Table 1). For the preparation of this protein, HEK293A cells were transiently transfected with pcDNA3.1(M23)AQP4, allowing the production of AQP4 as a 6-HIS-tagged protein. 72 h later, the cells were washed with sterile phosphate-buffered saline (PBS) and exposed to lysis buffer (10 mM Tris buffer pH7.5, 100 mM NaCl, 1 mM EDTA, 1 % Triton X-100 and complete protease inhibitor cocktail tablet) for 1 h at 4 °C. The lysate was thoroughly mixed by pipetting, subjected to repeated rounds of freezing and thawing, sonicated using a Sonopuls GM70 (Bandelin, Berlin, Germany), and finally passaged through a 23 gauge needle. Ni NTA-Agarose Superflow (Qiagen) was then used for the purification of AQP4 following the instructions of the manufacturer. Briefly, Ni NTA beads were gently applied to a column, washed with 5 volumes of wash buffer (20 mM Tris/125 mM NaCl) prior to applying the lysate mix diluted 1:1 in wash buffer containing 1 % Triton X-100. Following washing steps with 10 volumes of wash buffer containing 1 % Triton X-100, we were washing the column with 10 volumes of washing buffer containing 0.1 % Triton X-100. Subsequently, we eluted AQP4 in 20 mM Tris/125 mM NaCl/0.1 % Triton X-100/600 mM Imidiazole. Following dialyses against PBS we used AQP4 in a concentration of 1 mg/ml in PBS/0.1 % Triton X-100. The eluted AQP4 protein was confirmed by Western blot (data not shown). In specificity tests, also recombinant human MOG_1–125_ was used, which was essentially produced and purified as described [3]. The MOG_35–55_-specific T cells used were raised against rat/mouse MOG_35–55_ (Sigma).

### Immunization and T cell line preparation

The animals were subcutaneously immunized with 100 µl of a 1:1 mixture of the relevant antigen (stock 2 mg/ml) in Freund’s incomplete adjuvans supplemented with 4 mg/ml mycobacterium tuberculosis H37Ra. 9–11 days after the immunization, the animals were killed. At this point, they were all clinically healthy and did not show any evidence for inflammation of the CNS or of peripheral organs. The lymph nodes draining the immunization site were removed, and peptide-specific T cell lines were established as described [32, 33].

### Isolation of naïve T cells

The naïve T cells tested derived from the spleen of an adult Lewis rat housed under specific pathogen-free conditions. The spleen was processed to a single cell suspension, and contaminating erythrocytes were removed by incubation of the cells for 5 min in hypotonic salt solution (0.15 M NH_4_Cl, 1 mM KHCO_3_, 0.1 mM Na_2_EDTA) pH 7.4.

### Preparation of T cells for immunocytochemistry

T cells were embedded in HistoGel (Thermoscientific, Cheshire, UK) according to the manufacturer’s instructions and subsequently fixed with 4 % paraformaldehyde for 24 h. The HistoGel blocks were then processed for immunohistochemical analysis as detailed [2, 7].

### Characterization of T cell lines

#### Specificity tests

Specificity was determined in T cell proliferation assays, using 96 well plates. 5 × 10^5^ AQP4-peptide-specific T cells were cocultured in triplicates with 1 × 10^6^ thymic antigen presenting cells in the absence of externally added antigen or in the presence of irrelevant CNS antigens (i.e. myelin basic protein or unrelated AQP4-peptides; 10 µg/ml final) as negative controls, of the peptide against which the cell line was established as specific antigen (10 µg/ml final), or of concanavalin A (2.5 µg/ml final) as positive control [9, 11].

The human M23 AQP4 preparation had a protein concentration of 1 mg/ml in phoshate-buffered saline/0.1 % Triton X-100. To avoid the toxic effects of Triton X-100, we diluted the antigen preparation 1:100 with PBS and coated it in 100 µl aliquots over night at 4 °C onto flat-bottom 96-well plates. On the next morning, the coating solution was drained from the plates, and each well was washed gently and briefly with 100 µl culture medium containing 1 % rat serum. Then, 1 × 10^6^ thymic antigen presenting cells in 100 µl medium were cultured in these plates for 8 h at 37 °C prior to the addition of T cells. Exactly the same procedure was applied to recombinant human MOG_1–125_.

The cells were cultured for 48–72 h. For the final 18–24 h in culture, [^3^H]-thymidine was added to reveal de novo DNA synthesis during the S-phase of the cell cycle of activated T cells.

#### Analysis of surface marker expression by flow cytometry

For staining, the cells were incubated for 30 min at room temperature with antibodies against rat CD4 (W3/25, mouse monoclonal, Serotec) or mouse IgG1 (Dako, Glostrup, Denmark; isotype matched control antibody). After washing, the cells were incubated for 30 min at room temperature for 30 min with polyclonal Alexa488-labeled goat anti-mouse IgG (Jackson ImmunoResearch). For staining of the αβ-T cell receptor (TCR), PE-labeled mouse anti-αβTCR (eBioscience, San Diego, CA) was used. All antibodies were used in a dilution of 1:100 in stain buffer (PBS/10 % fetal calf serum/1 mM EDTA).

#### Quantitative real-time polymerase chain reaction (qPCR) for the detection of transcripts for IFN-γ and IL-17

RNA was purified from freshly activated T cell blasts using the RNeasy Plus Mini Kit (Qiagen GMbH, Hilden, Germany). Genomic DNA was removed and the RNA transcribed to cDNA as described [16]. qPCR was performed using the SsoAdvanced Universal SYBR Green Supermix (Bio-Rad) according to the manufacturer’s instructions, using a 10 µl reaction mixture [5 µl SsoAdvanced Universal SYBR Green Supermix, 0.2 µl forward primer (10 pmol/µl), 0.2 µl reverse primer (10 pmol/µl), 3.6 µl double-distilled H_2_O, 1 µl DNA template] in a StepOnePlus system (applied biosystems). The following primers were used: IL-17 forward: 5′-TACCAGCTGATCAGGACGAG-3′; IL-17 reverse: 5′-CATCAGGCACATGGATGGAA-3′; IFN-γ forward: 5′-ATTCATGAGCATCGCCAAGTTC-3′; IFN-γ reverse: 5′-TGACAGCTGGTGAATCACTCTGAT-3′; GAPDH forward: 5′-CCGAGGGCCCACTAAAGG-3′; GAPDH reverse: 5′-ATGGGAGTTGCTGTTGAAGTCA-3′. For qPCR, an initial denaturation step (95 °C, 30 s) was followed by 40 cycles of denaturation (95 °C, 15 s) and annealing/extension (60 °C, 1 min). The absence of unspecific amplification was determined by melt curve analysis. All reactions were run in triplicates.

### Induction of experimental autoimmune encephalomyelitis (EAE) and experimental autoimmune neuromyelitis optica (ENMO)

**Fig. 2:**
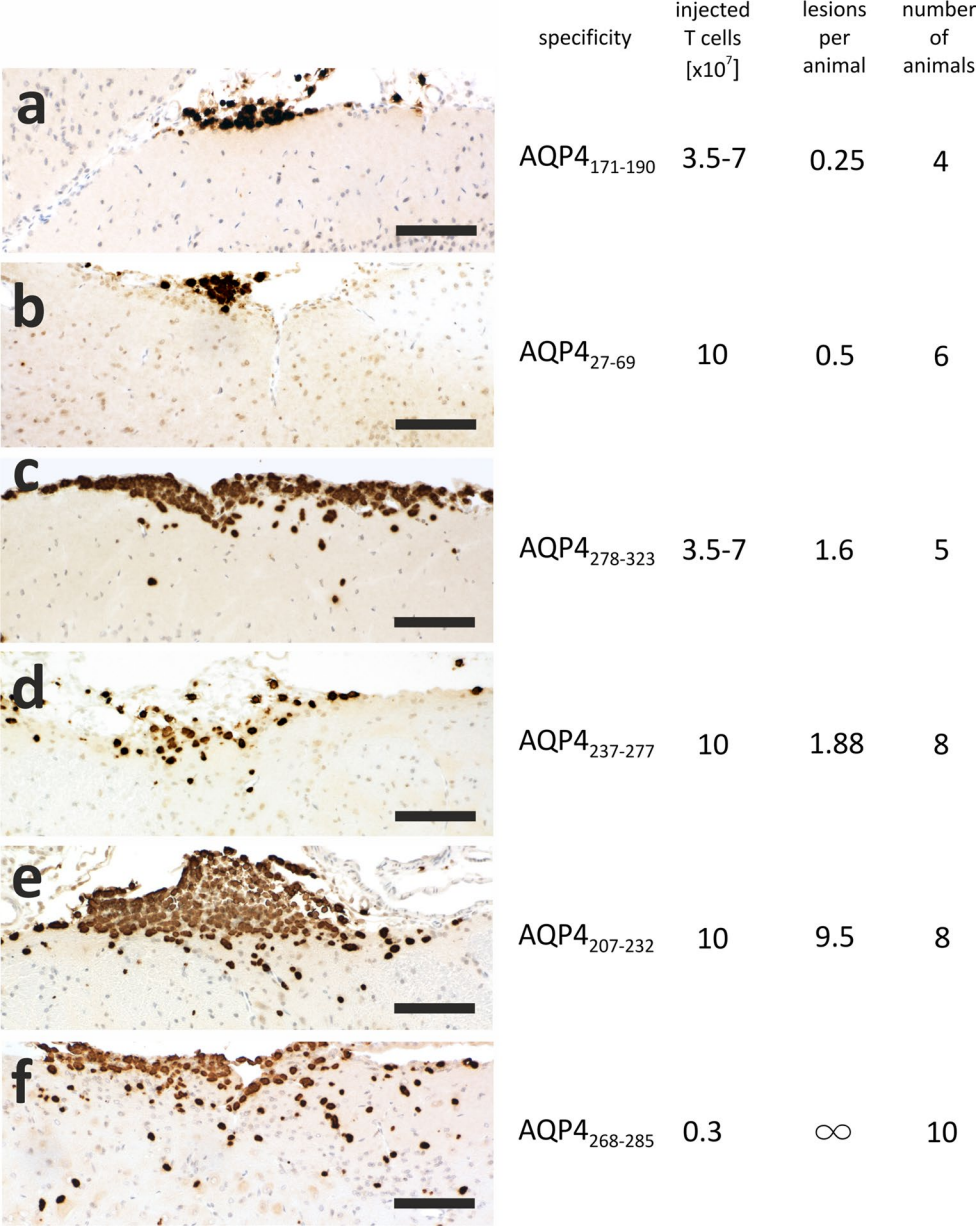
Inflammatory brain lesions after injection of aquaporin 4 peptide-specific T cells. Brain sections with lesions induced by AQP4_171–190_ (**a**), AQP4_27–69_ (**b**), AQP4_278–323_ (**c**), AQP4_237–277_ (**d**), AQP4_207–232_ (**e**), and AQP4_268–285_-specific T cells (**f**) were stained with anti-CD3 antibodies to reveal T cells (*brown*) and hematoxylin to show nuclei (*blue*). *Bars* 100 µm. Also shown are the average numbers of T cells which had to be injected in order to reliably get lesions, the average number of lesions per animal and the number of animals injected

EAE was induced in Lewis rats by intraperitoneal transfer of activated AQP4-peptide-specific T cells. The numbers of T cells transferred for each T cell line is shown in Fig. 2. The animals were monitored daily for weight loss and clinical signs of EAE, which was scored according to the following scheme: 0: healthy; 0.5: partial loss of tail tonus; 1: complete loss of tail tonus; 2: hind limb weakness, unsteady gait; 3: hind limb paralysis (this was for ethical reasons set as endpoint for EAE experiments). The animals were killed by an overdose of CO_2_ on days 5/6 after T cell-transfer, and perfused with 4 % phosphate-buffered paraformaldehyde (PFA). Brains and spinal cords were dissected, immersed for another 18–24 h in PFA and embedded in paraffin for histological analysis. For the induction of ENMO, AQP4-peptide-specific T cells were injected intraperitoneally on day 0, followed by an intraperitoneal injection with 1 ml phosphate-buffered saline (PBS) containing either 10 mg NMO-IgG or 10 mg normal human IgG on day 5. Also these animals were killed on day 7 after T cell-transfer with CO_2_, and perfused with 4 % PFA.

### Antigen injections into the cisterna magna

On day 5 after EAE induction by T cell transfer, the animals were anesthetized with Ketanest S/Rompun. Afterwards, the neck of the animals was flexed, and the skin was cut open to expose the atlanto-occipital membrane, which was then punctured with a thin glass capillary. When the entry of clear cerebrospinal fluid into the capillary indicated the accuracy of the injection site, 5 µl of AQP4 peptides (dissolved in a concentration of 2 mg/ml in RPMI), or of RPMI alone were injected slowly. Afterwards, the capillary was withdrawn and the skin was closed with wound clips. 24 h later, the animals were killed with CO_2_ and perfused with 4 % PFA. Brains and spinal cords were processed for immunohistochemical analysis as detailed [2, 7].

### Immunohistochemistry

All stainings were done essentially as described [2, 7] using the mouse monoclonal antibody ED1 (to stain macrophages and activated microglia; Serotec, Germany), rabbit polyclonal antibodies against CD3 (to stain T cells; NeoMarkers, Fremont, USA), rabbit polyclonal antibodies against AQP4 (to stain astrocytes; Sigma, Germany), rabbit polyclonal or mouse monoclonal antibodies against glial fibrillary acidic protein (GFAP; from Dako, Denmark, or NeoMarkers, respectively), anti-human immunoglobulin (biotinylated donkey; polyclonal; Amersham, UK), anti-rat immunoglobulin (biotinylated donkey; polyclonal; Jackson ImmunoResearch), and anti-complement C9 (rabbit polyclonal [29]).

For double immunostainings of proliferating cell nuclear antigen (PCNA, mouse, clone PC10, DAKO) and CD3 (rabbit polyclonal; NeoMarkers, Fremont, USA), sections were steamed in citrate buffer for 30 min. After incubation with mouse anti-proliferating cell nuclear antigen (PCNA, clone PC10, DAKO, 1:10 000) over night at 4 °C, the sections were incubated with alkaline-phosphatase-labeled anti-mouse antibodies (Jackson ImmunoResearch, West Grove PA, USA, 1:200) for 1 h at RT and developed with Fast Blue (FB, Sigma, Germany) substrate. Then the sections were incubated with rabbit anti-CD3 (polyclonal; NeoMarkers, Fremont, USA, 1:2000) over night at 4 °C. After incubation with biotinylated anti-rabbit antibodies (1:2000) for 1 h at RT and enhancement with CSA (1:1000) for 20 min, avidin-peroxidase (Sigma) was applied and the sections were developed with aminoethyl carbazole (AEC, Sigma).

### Quantitative evaluation of immunostained sections

Quantification was done by using a morphometric grid. To determine the extent of T cell infiltration, the number of CD3+ cells was determined in 3 different areas: within in the meninges, within the superficial parenchyma (<100 µm distance from the meninges) and within the deep parenchyma (>100 µm distance from the meninges).

### Statistical evaluation

Statistics were calculated with the IBM SPSS Statistics 21. The Mann–Whitney (Wilcoxon) *W* test (comparison of medians) was used in all cases. For multiple comparisons, Bonferroni correction was used.

## Results

### All AQP4 epitopes suitable for binding to Lewis rat MHC class II (RT1.B^L^) give rise to antigen-specific T cell responses

We used peptides spanning AQP4 epitopes previously identified as potential binders to RT1.B^L^ of Lewis rats [32] for the immunization of Lewis rats. Although all animals remained clinically healthy upon immunization, they mounted T cell responses against all of the peptides used. Consequently, different peptide-specific T cell lines could be established by alternating cycles of antigen-specific T cell activation and IL-2-driven T cell propagation (Table 1) which constrains the generation of T_H_17 cells [18]. These cells were CD4^+^, and expressed the αβ T cell receptor (suppl. fig  1). All of these cell lines showed a dominant expression of IFN-γ over IL-17 (suppl. fig 2), and therefore belonged to the T_H_1 subset of cells. They were all responsive to their specific peptides, but the AQP4_27–69_- and AQP4_278–232_-specific T cell lines did not reach a stimulation index >2 (suppl fig. 3).

We tested the encephalitogenic potential of these cells, i.e. their ability to induce CNS inflammation, by their transfer into naïve Lewis rats.

### AQP4-peptide-specific T cell lines vary in encephalitogenicity and ability for parenchymal infiltration

The different AQP4-peptide-specific CD4^+^ T cells varied in their ability to induce CNS inflammation and could be grouped into weak, medium, and strongly encephalitogenic lines. Weak encephalitogenicity was observed upon transfer of T cells with specificity for AQP4_171–190_, AQP4_27–69_, AQP4_278–323_, and AQP4_237–277_. Even after transfer of as many as 10 × 10^7^ T cells/animal, these lines were just able to yield an average of less than 2 lesions per rat. These lesions were essentially located within the meninges, and only very few T cells entered the CNS parenchyma (Fig. 2). The recipient animals did not show any symptoms of clinical disease, in line with this weak histological evidence of CNS inflammation. Medium encephalitogenicity was observed with T cells specific for AQP4_207–232_ containing the epitope PAVIMGNWE. On average, transfer of these cells lead to ~10 lesions per animal, distributed along the entire neuraxis. However, these cells only barely infiltrated the CNS parenchyma. Instead, they piled up in the meninges, resulting in up to 10 layers of T cells on top of each other (Fig. 2). Although there was more histological evidence of CNS inflammation than seen with the AQP4 peptide-specific T cells described above, we still did not see signs of clinical disease. Strong encephalitogenicity was observed in T cells specific for AQP4_268–285_.

### AQP4_268–285_-specific T cells are highly encephalitogenic

**Fig. 3:**
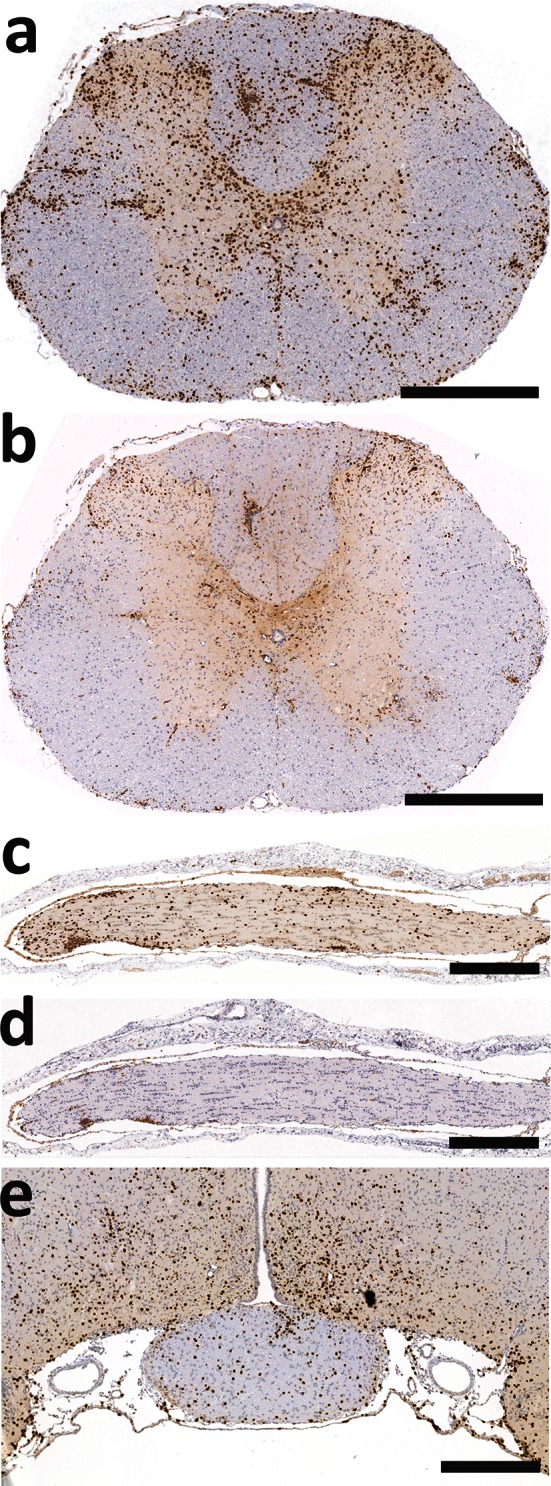
Inflammation of spinal cord and optic nerve provoked by AQP4_268–285_-specific T cells. Shown here are cross sections of thoracic spinal cord (**a**, **b**), longitudinal sections of the optic nerve (**c**, **d**) and a coronal section of the brain at the level of the optic chiasm (**e**), reacted with antibodies against CD3 to reveal T cells (**a**, **c**, **e**, *brown*) and with the antibody ED1 to show activated microglia/macrophages (**b**, **d**, *brown*). Counterstaining was done with hematoxylin to reveal nuclei (*blue*). *Bars* 1 mm

**Fig. 4:**
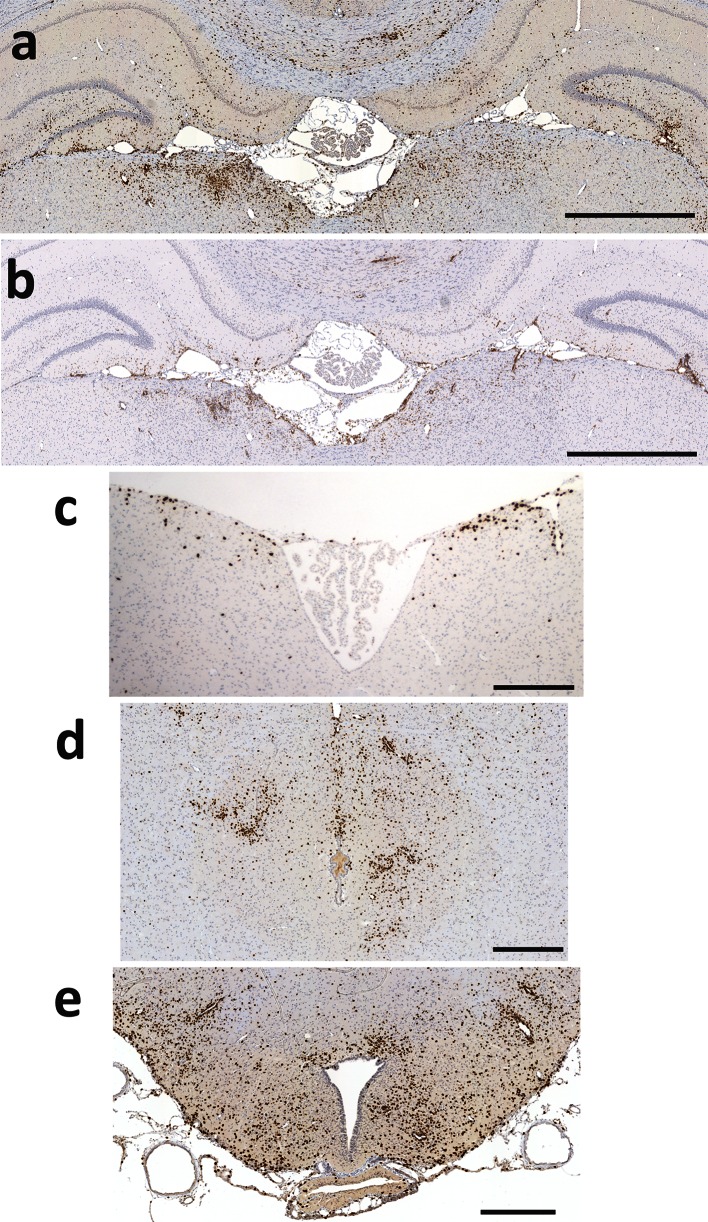
Inflammation of the brain provoked by AQP4_268–285_-specific T cells. Shown here are coronal sections of the brain at the level of the 3rd ventricle/hippocampus (**a**, **b**), the 4th ventricle/medulla (**c**), midbrain/periaqueductal gray (**d**), and basal hypothalamus/eminentia mediana (**e**) reacted with antibodies against CD3 to reveal T cells (**a**, **c**, **d**, **e**, *brown*) and with the antibody ED1 to show activated microglia/macrophages (**b**, *brown*). Counterstaining was done with hematoxylin to reveal nuclei (*blue*). The inflammatory sites shown are representative for 5 animals analyzed. *Bars* 1 mm

While for all other cell lines, 3.5-10 × 10^7^ T cells had to be transferred to produce inflammatory CNS lesions, as few as 0.03 × 10^7^ AQP4_268–285_-specific T cells/animals sufficed to do so. For all analysis described below, 3 × 10^6^ AQP4_268–285_-specific T cells/animals were used. These cells caused CNS inflammation along the entire neuraxis, i.e. in all levels of the spinal cord, throughout the optic nerve, and in the entire brain (Figs. 3, 4). “Hotspots” for inflammatory lesions were areas around the 3rd and 4th ventricle, in the hippocampus, in the periaqueductal gray, in cerebellum and medulla, and in the spinal cord in dorsal horns and central gray matter (Figs. 3, 4). Lesions at similar sites in the brain were also seen after transfer of T cells with other CNS antigen specificities like S100β or MBP (suppl fig. 4). However, the massive dominance of T cell infiltrates in spinal cord gray matter seen with AQP4_268–285_-specific T cells was unique for these cells (suppl. fig 4). The CNS lesions provoked by AQP4_268–285_-specific T cells were essentially T cell-dominated with little microglia activation/macrophage recruitment (Figs. 3, 4), and caused weight loss with partial loss of tail tonus as sole clinical symptom. Some AQP4_268–285_-specific T cells were found in the meninges, but a much larger proportion of these cells was able to infiltrate the CNS gray and white matter parenchyma than seen with any other AQP4-peptide-specific T cell line before (Figs. 3, 4).

### AQP4_207–232_- and AQP4_268–285_-specific T cells differ in their extent of activation within the CNS

**Fig. 5:**
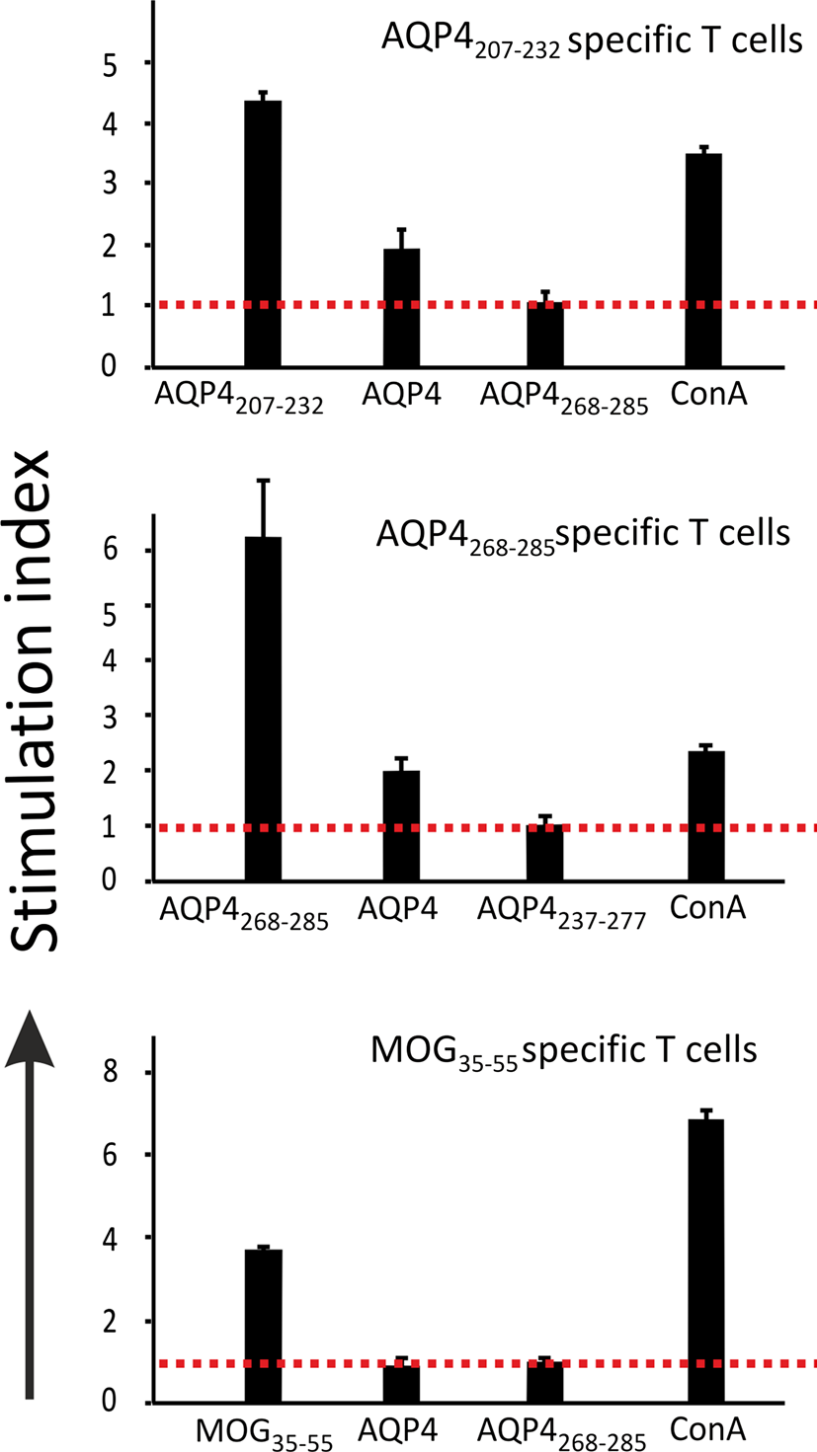
AQP4 is naturally processed to peptides recognized by AQP4_207–232_- and AQP4_268–285_-specific T cells. 5 × 10^5^ AQP4-peptide-specific T cells were cocultured in triplicates with 1 × 10^6^ thymic antigen presenting cells in the absence of externally added antigen or in the presence of irrelevant CNS antigens (i.e. AQP4_268–285_ for AQP4_207–232_-specific T cells or AQP4_237–277_ for AQP4_268–285_-specific T cells; 10 µg/ml final) as negative controls, of the peptide against which the cell line was established as specific antigen (10 µg/ml final), full-length human M23 AQP4 (well coated with 10 µg protein) or of concanavalin A (2.5 µg/ml final) as positive control. MOG_35–55_-specific T cells were used as negative control to exclude any unspecific effects of the M23 AQP4 coating. T cell activation was evidenced by the incorporation of ^3^H-thymidine and measured in counts per minute (cpm). Shown here are the stimulation indices (cpm of peptide or protein-exposed cells over cpm of cells cultured in the presence of the irrelevant CNS antigen) ±standard deviation. Data are representative of several independently performed tests

**Fig. 6:**
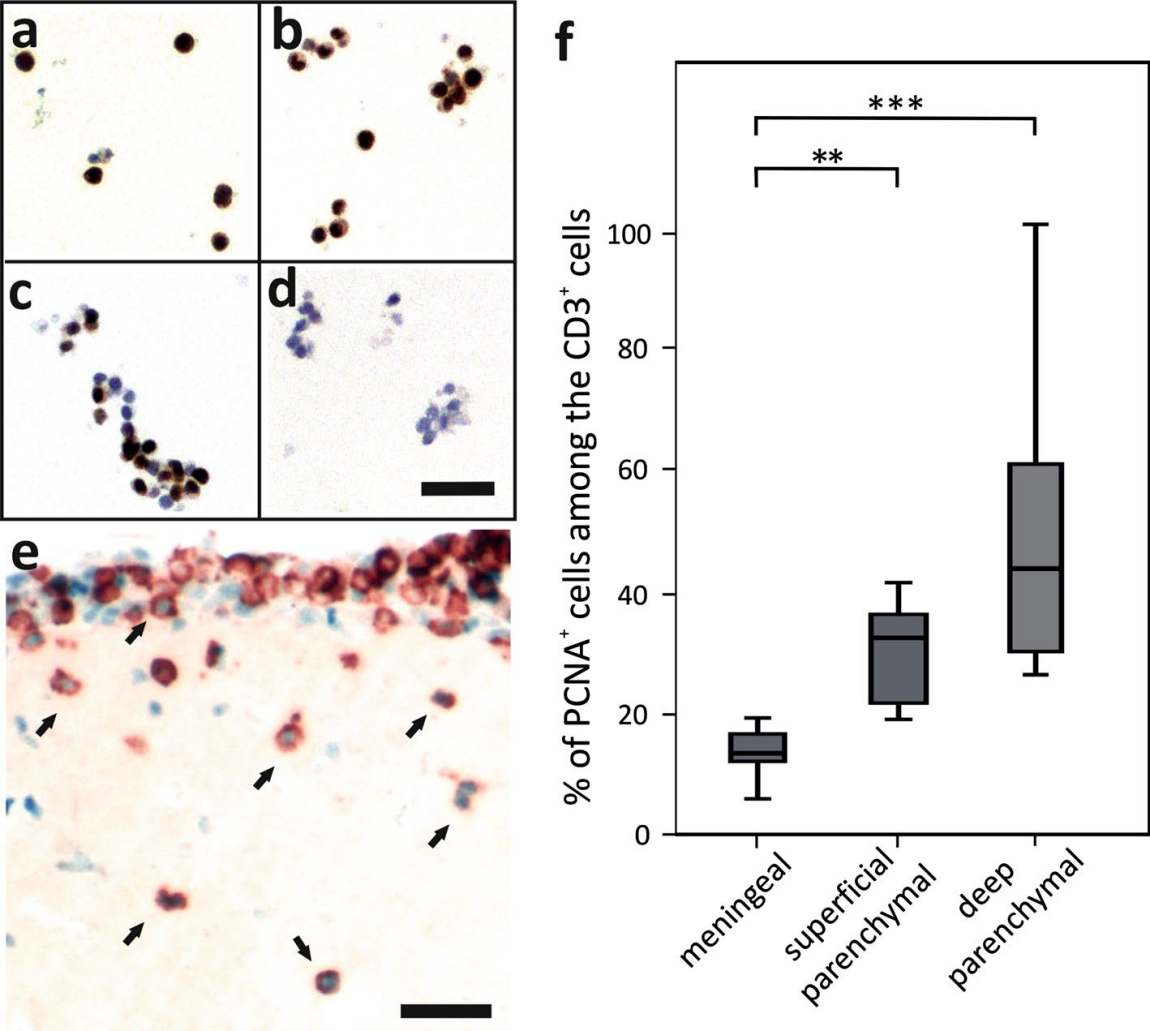
Activation of AQP4 peptide-specific T cells in vitro and in vivo. To show that the expression of the proliferating cell nuclear antigen (PCNA) depends on T cell activation, AQP4_207–232_-specific T cells were activated with “their” specific peptide in the context of RT1.B^L^, were then purified by density gradient centrifugation and cultured in IL-2 containing medium. Immediately after density gradient centrifugation (day 0, **a**), and after additional 2 (**b**), 4 (**c**) and 6 (**d**) days in culture, the cells were stained with antibodies against the proliferating cell nuclear antigen (PCNA, *brown*) and counterstained with hematoxylin (*nuclei blue*). **e** Lesion in the basal hypothalamus induced by AQP4_268–285_-specific T cells, stained with PCNA (*blue*) and CD3 (*red*). The* arrows* point to double-positive T cells which immigrated the CNS parenchyma. Please note that there is also PCNA staining of activated microglia/macrophages. **f** The percentage of CD3^+^PCNA^+^ cells in the CD3^+^ T cell pool was determined within the meninges proper, within the superficial parenchyma (<100 µm distance from the meninges) and within the deep parenchyma (>100 µm distance from the meninges), using a morphometric grid and ×25 magnification. We counted the cells in 3 different lesions/rat brain from 7 different rats after EAE induction with AQP4_268–285_-specific T cells. The differences between the lesion areas were significant (Mann–Whitney exact *U* test and Bonferroni–Holm correction; *p* = 0.0015 (**) for meningeal/superficial-parenchymal cells, *p* = 0.00015 (***) for meningeal/deep parenchymal cells). *Scale bars* 25 µm

Why are AQP4_268–285_-specific T cells able to deeply immigrate into the CNS parenchyma, while all other AQP4_peptide_-specific T cells studied so far pile up in the meninges? To address this question, we made T cell proliferation assays to analyze whether antigen presenting cells can process full-length AQP4 to produce and present T cell epitopes to AQP4_207–232_-, and AQP4_268–285_-specific T cells. We observed that these T cells became activated and proliferated in the presence of AQP4 and syngenic antigen presenting cells (Fig. 5; suppl fig. 5), indicating that the epitopes recognized arise from naturally processed AQP4. We next focused on AQP4_268–285_-specific T cells and on AQP4_207–232_-specific T cells as “prototype” for all other AQP4_peptide_-specific T cells and studied the activation of AQP4_268–285_- and AQP4_207–232_-specific T cells in the CNS. It was not possible to re-isolate and characterize these cells by FACS analysis, since AQP4_207–232_-specific T cells had to be used in extremely high cell numbers for EAE induction (Fig. 2) and caused only few lesions of small size. Instead, we studied T cell activation by immunohistochemistry, using double stainings with CD3 as T cell marker, and with the proliferating cell nuclear antigen PCNA as activation marker. To ensure that PCNA is a reliable marker for T cell activation, we first tested the anti-PCNA antibody in vitro, using T cells cultured for different lengths of time after their antigen-specific activation. We observed, that PCNA was detectable in 90 % of the T cells on day 0 after a 48 h-lasting T cell activation by antigen in the context of RT1.B^L^, in 80 % of the T cells on day 2, in 50 % of T cells on day 4, and became essentially undetectable (<1 %) in T cells on day 6 (Fig. 6). We next evaluated the percentage of activated PCNA^+^ AQP4_268–285_- specific T cells in the total CD3^+^ T cell pool, depending on the depth of parenchymal infiltration. In medians, 14 % PCNA^+^ CD3^+^ T cells were found in the meninges, 32 % located in the superficial, and 44 % in the deep parenchyma. Cumulatively, these data showed that AQP4_268–285_-specific T cells are much better activated in the CNS than AQP4_207–232_-specific T cells. What restricts the activation of AQP4_207–232_-specific T cells?

### The amount of available AQP4_207–232_-peptide limits CNS infiltration by AQP4_207–232_-peptide-specific T cells

**Fig. 7:**
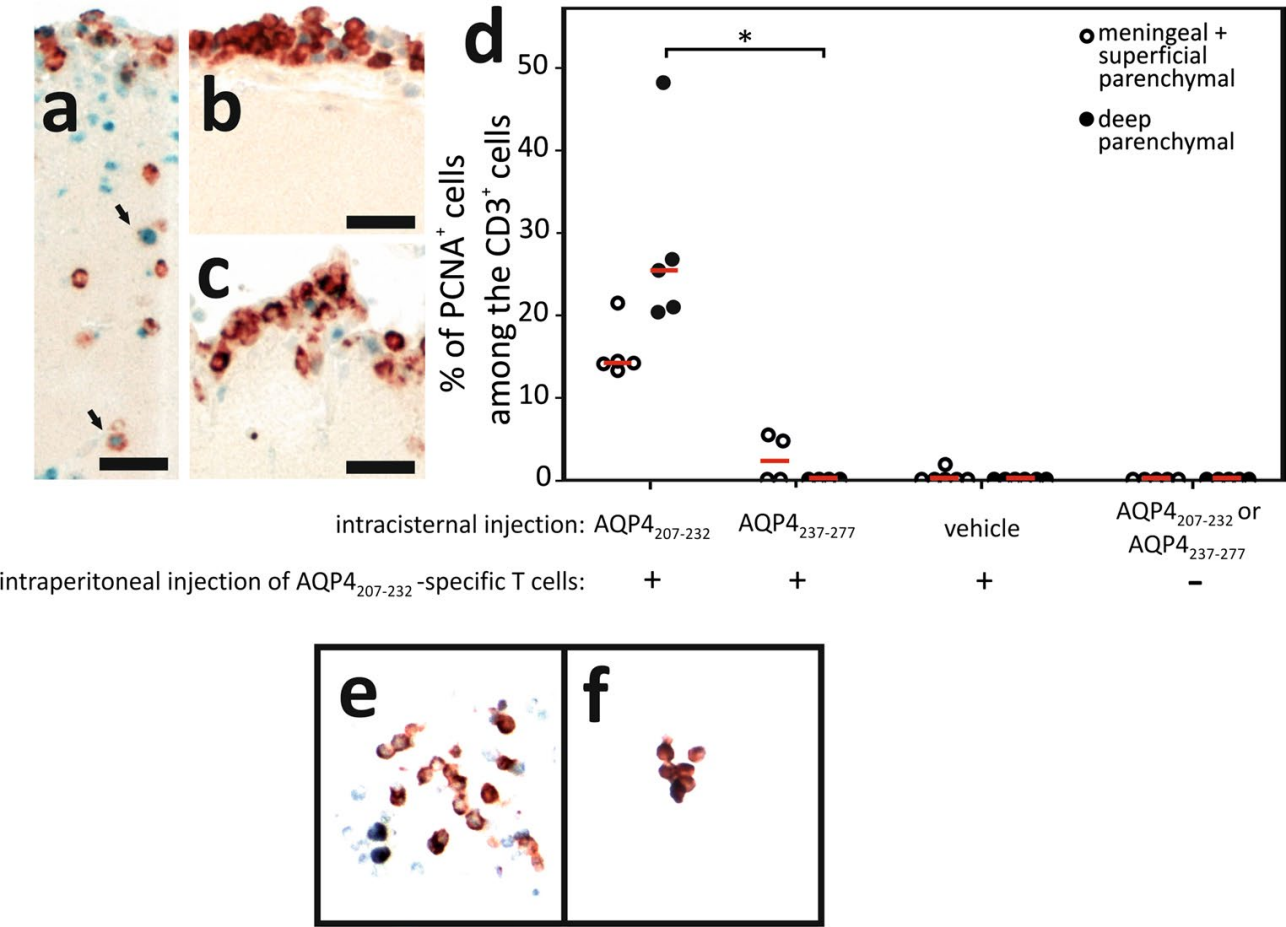
Immigration and activation of AQP4_207–232_–specific T cells into the CNS parenchyma upon intracisternal antigen injection. Tissue sections of Lewis rats which had been injected with AQP4_207–232_–specific T cells to induce EAE. On day 5 after T cell transfer, the animals received an injection into the cisterna magna of either AQP4_207–232_ as specific antigen (**a**), AQP4_237–277_ as irrelevant control peptide (**b**), and vehicle (**c**), and were sacrificed 24 h later for histological analysis. Shown here are dorsal medullas, stained with anti-CD3 antibodies to identify T cells (*brown*), and with anti-PCNA antibodies to reveal activation of cells (blue nuclei). *Scale bars* 100 µm. **d** Quantification of the percentage of PCNA^+^ cells among CD3^+^ T cells in the dorsal medullas, using a morphometric grid for counting and a ×10 magnification. Shown here is the percentage of PCNA^+^ T cells (>100 µm distance from the meninges) in the total CD3^+^ T cell pool at this site after EAE induction with AQP4_207–232_–specific T cells and intracisternal injection of either AQP4_237–277_ (*n* = 4), AQP4_207–232_ (*n* = 5), or vehicle (*n* = 6), or in naïve Lewis rats after intracisternal injection of AQP4_237–277_ or AQP4_207–232_. The differences between the treatments with the unspecific (AQP4_237–277_; *n* = 4) and the specific (AQP4_207–232_; *n* = 5) peptide were significant (Mann–Whitney exact *U* test; *p* = 0,016). Naïve T cells (**e**) or naïve T cells cultured in the presence of AQP4_207–232_ and spleen-derived antigen presenting cells (**f**) do not express PCNA [anti-CD3 antibodies (*red*) and anti-PCNA antibodies (*blue*) were used]

To test whether low amounts of AQP4_207–232_ preclude efficient T cell activation and subsequent parenchyma infiltration, we increased its concentration by injecting AQP4_207–232_ into the cisterna magna at the very onset of AQP4_207–232_-peptide-specific T cell-mediated EAE [1, 42]. Intracisternal injections of an irrelevant AQP4 peptide (AQP4_236–277_) and of vehicle (=RPMI) into AQP4_207–232_-peptide-specific T cell-challenged rats, and of peptides AQP4_236–277_ and AQP4_207–232_ into naïve rats served as controls. The animals were killed 24 h later for immunohistochemical analyses. When AQP4_207–232_ was injected into the cisterna magna, 33 % of the T cells migrated into the deep parenchyma, while this was the case for only 7.5 % of the T cells when the irrelevant AQP4_236–277_ peptide has been used (data not shown). Moreover, 25 % of the CD3^+^ T cells found in the deep parenchyma had an activated phenotype, as evidenced by the expression of PCNA (Fig. 7). The most likely source of these activated T cells are the AQP4_207–232_-specific T cells which had been injected into these animals to induce EAE, since we did not see PCNA expression in naïve T cells cultured without any antigen, or cultured in the presence of AQP4_207–232_ and splenic antigen presenting cells (Fig. 7).

Hence, T cell activation and subsequent parenchymal infiltration by AQP4_207–232_-peptide-specific T cells can be increased by injections of AQP4_207–232_ into the cisterna magna.

### In the presence of NMO-IgG, the numbers of pathogenic T cells determine location and size of astrocyte-destructive lesions

**Fig. 8:**
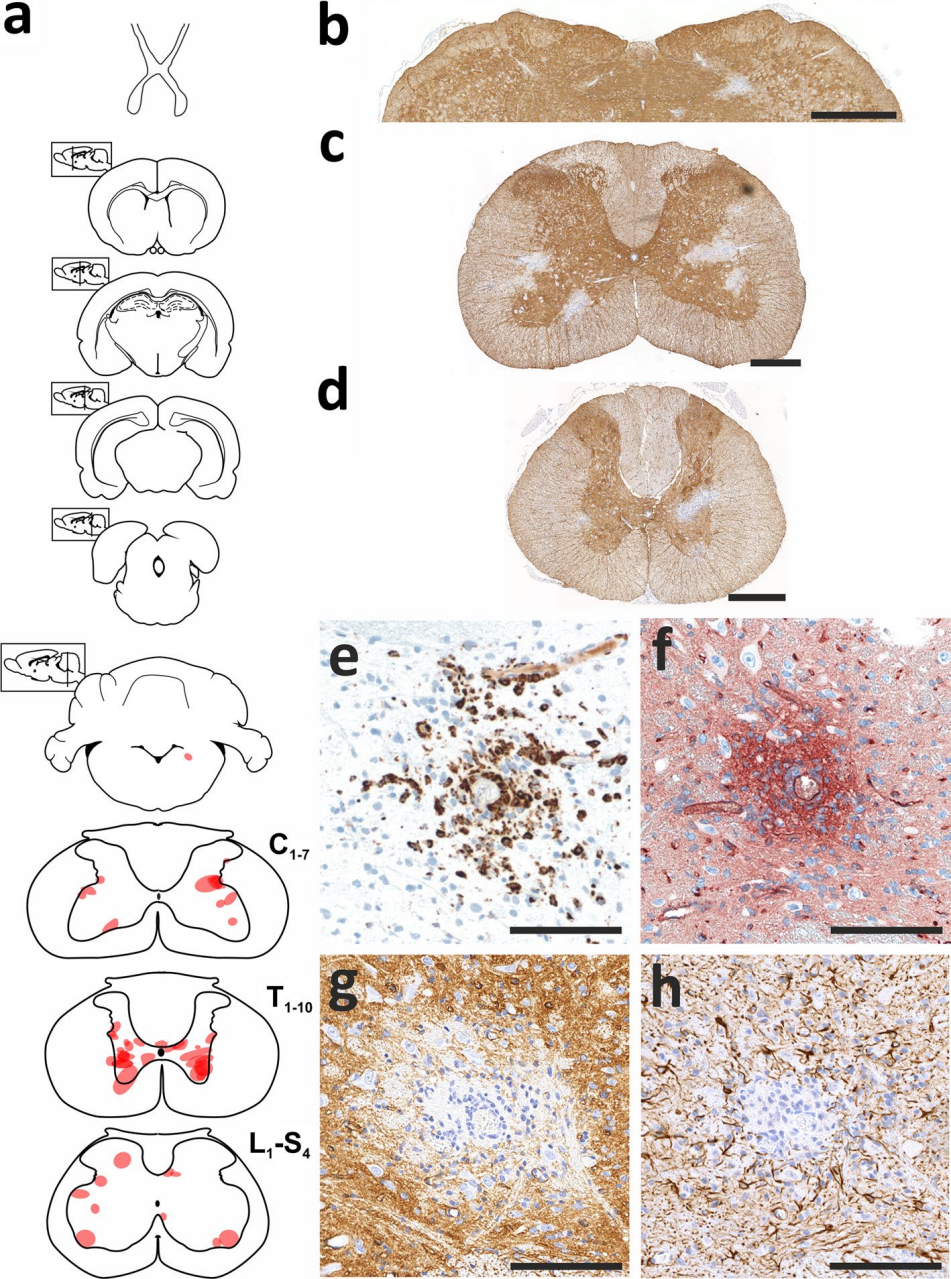
In the presence of NMO-IgG, low numbers of AQP4_268–285_-specific T cells initiate astrocyte-destructive lesions almost exclusively in the spinal cord. **a** Distribution of lesions with AQP4 loss along the neuraxis, using schemes provided by Paxinos and Watson [28] as guide lines. Shown here are brain and spinal cord [cervical (C_1–7_), thoracal (T_1–10_) and lumbar/sacral (L_1_–S_4_)] sections of 3/5 animals, and the location of each lesion with AQP4 loss was projected in *red color* into the relevant scheme. Histological sections of the medulla (**b**), cervical (**c**) and thoracal (**d**) spinal cord sections are shown. These sections were reacted with anti-AQP4 antibodies to show the presence (*brown*) and the loss (*white*) of this protein. Counterstaining was done with hematoxylin to reveal nuclei (*blue*). *Bars* 1 mm (**b**) and 0.5 mm (**c**, **d**). One lesion is shown in consecutive sections reacted with ED1 to show the presence of activated microglia/macrophages (**e**, *brown*), with antibodies against C9 to show the rosette-like perivascular complement deposition typical for NMO lesions (**f**, *red*), against AQP4 (**g**, *brown*) and against GFAP (**h**, *brown*). The sections were counterstained with hematoxylin for blue nuclear staining, and the *bars* 125 µm

**Fig. 9:**
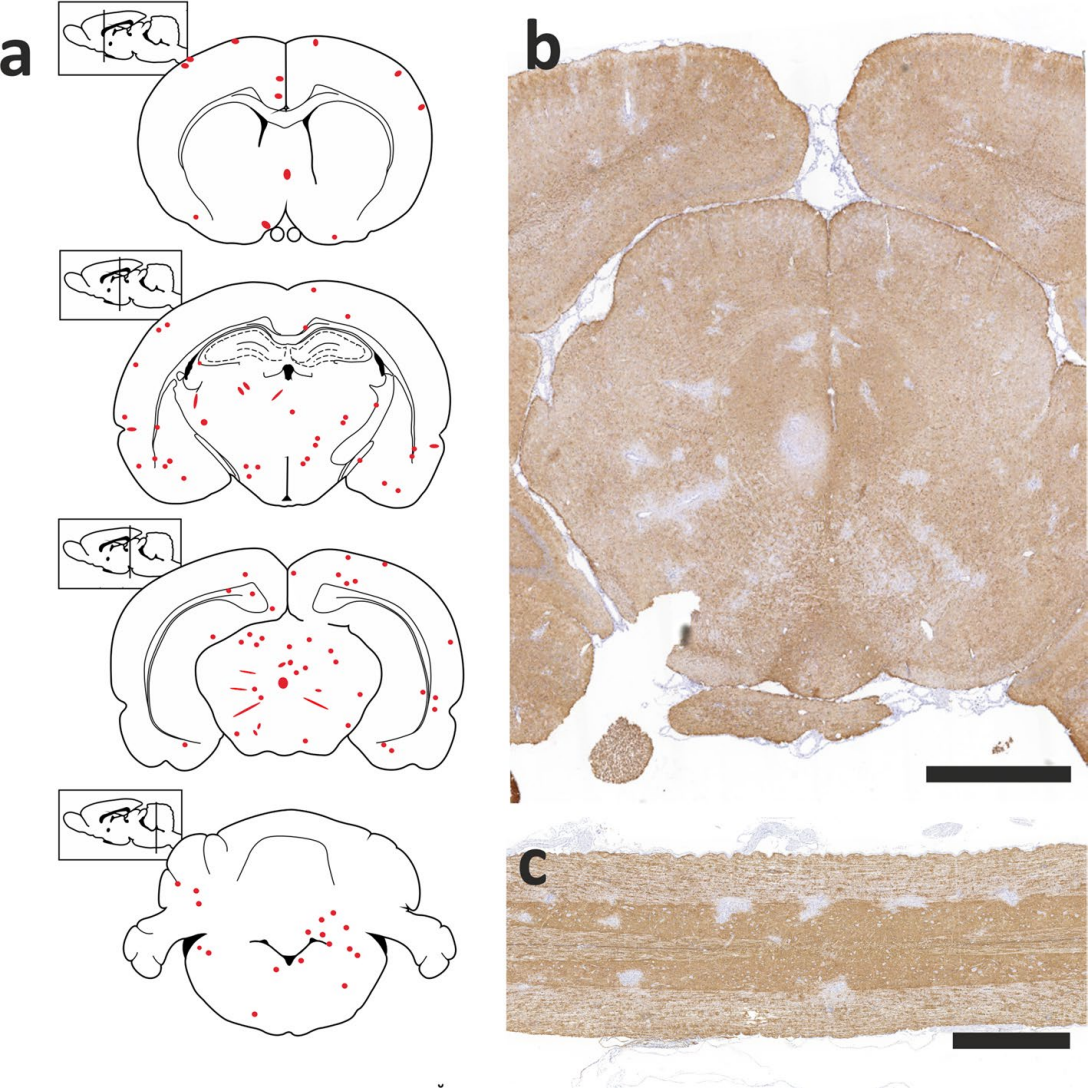
In the presence of NMO-IgG, high numbers of AQP4_268–285_-specific T cells initiate astrocyte-destructive lesions in spinal cord and brain. **a** Distribution of lesions with AQP4 loss throughout the brain, using schemes provided by Paxinos and Watson [28] as guide lines. Shown here are coronal sections of 2 rats, and the location of lesions with AQP4 loss was projected in *red color* into the relevant scheme. Coronal section cut at midbrain level (**b**) and longitudinal section of the spinal cord (**c**) were reacted with anti-AQP4 antibodies to show the presence (*brown*) and the loss (*white*) of this protein. Counterstaining was done with hematoxylin to reveal nuclei (*blue*). *Bars* 1.5 mm

We have shown above that AQP4_268–285_-specific T cells are sufficiently activated to immigrate the CNS parenchyma. Are they also sufficiently activated to allow the formation of astrocyte-destructive lesions in the presence of NMO-IgG? To address this question, we challenged Lewis rats with AQP4_268–285_-specific T cells and NMO-IgG, and studied their spinal cords, optic nerves, and brains by immunohistochemistry. When 3 × 10^6^ AQP4_268–285_-specific T cells were used in combination with 10 mg NMO-IgG, the animals showed loss of tail tonus (EAE score 1) at the day of sacrifice (suppl. fig 6), and lesions exhibiting loss of both AQP4 and GFAP reactivity almost exclusively confined to the spinal cords (54/55 lesions). These lesions were most frequently found in thoracic cord gray matter (51.8 %) than in cervical (22.2 %) or lumbar/sacral cord gray matter (18.5 %) (Fig. 8), reaching sizes up to 47,105 µm^2^. Astrocyte-destructive lesions were not seen in the optic nerves, and were only detected once in the dorsal medulla (1/55 lesions) (Fig. 8, suppl. fig 8). The outcome was different, when 2 × 10^7^ AQP4_268–285_-specific T cells were used together with 10 mg NMO-IgG. Then, lesions with AQP4 loss were not only seen in the spinal cord, reaching sizes up to 26,274 µm^2^, but also in the brain (Fig. 9). The optic nerves contained T cell infiltrates and activated microglia/macrophages, but did not show any evidence of AQP4 loss (suppl. fig 8). Lesions with AQP4 loss were also absent from optic nerves of 3-week-old rats injected with AQP4_268–285_-specific T cells and NMO-IgG (data not shown).

Hence, in the presence of NMO-IgG, low numbers of AQP4_268–285_-specific T cells initiate astrocyte-destructive lesions almost exclusively in spinal cord gray matter, while higher numbers of AQP4_268–285_-specific T cells trigger additional lesions with AQP4 and GFAP loss in the brain.

## Discussion

Since the discoveries of T cells in NMO lesions [21], and of NMO-IgG containing pathogenic AQP4-specific antibodies with the T cell-dependent immunoglobulin subclass IgG1 [19, 20], the question arose whether AQP4 specific T cells are only responsible for T cell help in antibody formation, or whether they can also participate in directing lesions to NMO-typical sites, and induce there astrocyte-destructive lesions in the presence of NMO-IgG. And indeed, all AQP4-specific T cells established so far from wildtype animals were only weakly encephalitogenic, targeted the wrong sites, and were essentially confined to the meninges, but only poorly infiltrated the CNS parenchyma [32]. To finally answer these questions, we used one model organism—Lewis rats—to raise T cell lines against all AQP4 epitopes predicted to bind to the MHC class II products (RT1.B^L^) of these animals [40], and to test the encephalitogenic potential of these cells in vivo. While all of these peptides could serve as antigens to produce stable CD4^+^ T_H_1 cell lines and were fully able to activate T cells in vitro, the vast majority of the resulting AQP4 peptide-specific T cells were only weakly or moderately encephalitogenic. Such cells had to be transferred in very high numbers into naïve animals, did not yield any clinical symptoms, and barely infiltrated the CNS parenchyma. However, one of the peptides used, i.e. AQP4_268–285_, was clearly different. It contains two overlapping epitopes (AQP4_271–279_ with the amino acid sequence QQTKGSYME, and AQP4_273–281_ with the amino acid sequence TKGSYMEVE), is the autoantigen of highly encephalitogenic T cell responses in Lewis rats, and gives rise to T cells which induce clinical symptoms, deeply immigrate the CNS parenchyma, and initiate large astrocyte-destructive lesions in the presence of NMO-IgG.

What did we learn from these AQP4_268–285_-specific T cells, and how close do we come to an animal model for NMO when we use them to induce CNS inflammation in combination with pathogenic NMO-IgG in Lewis rats?

1. AQP4_268–285_-specific CD4^+^ T cells are found in the normal healthy immune repertoire, can be readily activated upon immunization, and induce severe panencephalitis upon injection into naïve rats. Hence, AQP4_268–285_ is a true self-antigen in Lewis rats.
2. AQP4_268–285_-specific T cells can immigrate into the CNS parenchyma throughout the entire neuraxis, but are particularly frequent at sites described to be typical for NMO [31]: AQP4_268–285_-specific T cells cause myelitis with a strong involvement of the dorsal horns and central gray matter, optic neuritis, and encephalitis with profound infiltration around the 3rd and 4th ventricle and in the hippocampus, in the periaqueductal gray, in the cerebellum and in the medulla. These sites are also lesion sites in NMO patients with brain involvement [30, 43], which has initially been ascribed to the high AQP4 expression at these sites [31]. However, since we also see inflammation at these sites, when animals have been challenged with other CNS-antigen-specific T cells, this distribution of brain lesions is more likely to reflect the local action of other regulatory mechanisms for T cells and antibody-mediated processes.
3. AQP4_268–285_-specific T cells yield inflammatory lesions in which ~44 % of the deeply infiltrating T cells express PCNA as a sign of recent activation. Again, this is a crucial point, since T cell activation within the CNS is an important prerequisite for the formation of astrocyte-destructive lesions in the presence of NMO-IgG lesions [33], and since activated CD4^+^ T cells are found in NMO lesions [33]. It is tempting to speculate that this high level of PCNA expression might be due to reactivation within the CNS and not due to activation in vitro before transfer. A supportive, but certainly not definitive evidence for this speculation is the low number of proliferating AQP4_207–232_-specific T cells in the CNS, which is massively increased by additional injection of the respective peptide into the CSF. Unfortunately, however, we cannot formally prove whether or not antigen presenting cells in the CNS process antigens in a similar way as their counterparts in vitro do, and whether full-length AQP4 is cleaved by these cells to AQP4_207–232_ as efficiently in the CNS as it is in vitro.
4. High numbers of AQP4_268–285_-specific T cells target astrocyte-destructive lesions throughout the entire spinal cord, and also to the brain in NMO-IgG seropositive hosts. However, when present in low numbers, AQP4_268–285_-specific T cells target 98 % of all astrocyte-destructive lesions to cervical/thoracic spinal cord gray matter in NMO-IgG seropositive hosts. The preference of spinal cord is important, since NMO often presents with episodes of myelitis. Targeting of this site could result from higher levels of expression of AQP4 mRNA, protein, and large supramolecular aggregates in spinal cord and optic nerve relative to other regions of the brain [22], and in gray relative to white matter cord [7]. Higher antigen concentrations might then translate to better binding of NMO-IgG, to an enhanced availability of this antigen for local antigen presenting cells and subsequent T cell activation [24], and to an increased astrocytotoxicity of microglia/macrophages via complement- and antibody-mediated cellular mechanisms [33, 34].
  In combination with NMO-IgG, AQP4_268–285_-specific T cells also show a predilection for cervical/thoracic spinal cord, which are sites most often affected in NMO patients. Since this area is also most frequently targeted in the spinal cords of Lewis (LEW), LEW.1 N and LEW.1A rats with EAE provoked by the action of myelin oligodendrocyte glycoprotein (MOG)-specific antibodies and T cells [41], this might point to a gateway for autoreactive T_H_1 cells and antibodies to cross the blood–brain barrier at this site, possibly defined by regional neural activation [5].
  We do not know yet why in our current NMO model the optic nerves are spared from astrocyte-destructive lesions, although they contain numerous inflammatory T cells and activated microglia/macrophages. Most trivially, the formation of NMO-like lesions in the optic nerve could just simply be a rare event in ENMO provoked by AQP4_268–285_-specific T cells and NMO-IgG, and could become visible when higher numbers of animals are examined. Alternatively, also the genetic background of our animals might play a role, since there is, again, a striking resemblance to the MOG-induced EAE model described above. In the MOG-model, demyelinating spinal cord lesions formed in LEW.1 N, LEW.1A, LEW.1AV1, BN, and DA rats, but additionally in the optic nerves only in BN and DA rats [35]. This contribution of genetic background to disease phenotype could find its human correlate in the different, ethnicity-dependent frequencies of longitudinally extensive transverse myelitis seen at onset attack in 53 % of Caucasian vs. 33 % of Afro-Caribbean patients in an UK cohort of NMO patients [16].
5. Also low numbers of AQP4_268–285_-specific T cells can initiate large lesions with AQP4 loss. These findings recapitulate observations which have been made earlier in an EAE model using myelin basic protein-specific T cells with demyelinating anti-MOG antibodies [17], and suggest that the large, astrocyte-destructive lesions in NMO-IgG seropositive NMO patients could be provoked by the action of very few AQP4-specific T cells.
  In Lewis rats, AQP4_268–285_ is highly encephalitogenic. For the time being, we do not know yet whether intracellular AQP4 fragments also contain highly encephalitogenic antigens in humans, since their MHC could select different epitopes. Does this mean that AQP4-specific T cells recognizing weakly encephalitogenic AQP4 epitopes are irrelevant for NMO in Lewis rats or NMO patients? Probably not. While certain antigenic fragments might not be present in sufficient amounts to warrant T cell infiltration into the intact CNS, i.e. to trigger the very first NMO lesion, they might play a crucial role in the propagation of relapses, for example when antigens are released from necrotic, astrocyte-destructive lesions. Then, the liberated antigens might become available for local antigen presenting cells [13, 26], and provide the basis for the activation of naive T cells within the CNS in a process called epitope spreading [17]. At least in EAE and MS, this process underlies the shift of autoreactivity from primary initiating self-determinants, which invariably regress with time and might even become undetectable during periods of disease progression, to sustained secondary autoreactivity [37]. Considering the fact that AQP4 contains a large number of potential T cell epitopes, not only in mice and rats [12, 25, 32], but also in humans [6, 23] (Fig. 1), it is tempting to speculate that this might be a strong argument in favor of very early T cell vaccination, and a strong counter-argument for later T cell vaccination as a therapeutic option for NMO patients.

## Electronic supplementary material

Supplementary material Fig. 1: Surface markers of AQP4-peptide-specific T cell lines. Flow cytometry analysis of freshly activated, purified AQP4-peptide-specific T cell blasts revealed that they express CD4 and the αβ T cell receptor, as evidenced by the binding of the antibodies W3/25 and R73, respectively. Shown here are histograms of single stainings, and dotblots of double stainings. (TIFF 31,708 kb)

Supplementary material Fig. 2: Cytokine markers of AQP4-peptide-specific T cell lines. Freshly activated, purified AQP4-peptide-specific T cell blasts were tested by qPCR for their expression of interferon-gamma (IFN-γ) and interleukin-17 (IL-17) in relation to the house keeping gene glyceraldehyde-3-phosphate dehydrogenase (GAPDH). Normalized gene expression levels were calculated using the equation 2^−∆Ct^ = 2^−[ct (cytokine)−ct[GAPDH]^). Shown here are means +/− standard deviation of technical triplicates. (TIFF 16,130 kb)

Supplementary material Fig. 3: Recognition of the specific antigen by AQP4-peptide-specific T cell lines. 5 × 10^5^ AQP4-peptide-specific T cells were cocultured in triplicates with 1 × 10^6^ thymic antigen presenting cells in the absence of externally added antigen or in the presence of irrelevant CNS antigens (labeled with black arrow) as negative controls, of the peptide against which the cell line was established as specific antigen (10 µg/ml final; labeled with white arrow), or of concanavalin A (2.5 µg/ml final) as positive control. T cell activation was evidenced by the incorporation of ^3^H-thymidine and measured in counts per minute (cpm). Shown here are the stimulation indices (cpm of peptide or protein-exposed cells over cpm of cells cultured in the presence of the irrelevant CNS antigen) +/− standard deviation. (TIFF 23,600 kb)

Supplementary material Fig. 4: Location of inflammatory CNS lesions provoked by other CNS antigen-specific T cells. Infiltration of T cells specific for MBP (a,d,g,j,m), S100β (b,e,h,k,n), and MOG (c,f,I,l,o) along the neuraxis of Lewis rats. Shown here are sections of thoracic spinal cord (a-c; the dotted line indicates the gray/white matter border), optic nerve/chiasm (d-f), and brain at the level of the 3rd ventricle (g-i), midbrain/periaqueductal gray (j-l), and basal hypothalamus/eminentia mediana (m-o) reacted with antibodies against CD3 to reveal T cells (brown). Counterstaining was done with hematoxylin to show nuclei (blue). The inflammatory sites depicted are representative for CNS inflammation caused by T cells with the indicated CNS antigen-specificity. (TIFF 27,184 kb)

Supplementary material Fig. 5: AQP4-specific T cells do not respond to recombinantly expressed and purified MOG, while MOG-specific T cells do. 5 × 10^5^ AQP4_207–232_-peptide-specific or MOG_35–55_-peptide-specific T cells were cocultured in triplicates with 1 × 10^6^ thymic antigen presenting cells in the presence of irrelevant CNS antigen (AQP4_268–285_ for AQP4_207–232_-specific T cells, or AQP4_207–232_ for MOG_35–55_-specific T cells; 10 µg/ml final) as negative control, of the specific peptide against which the cell line was established as specific antigen (AQP4_207–232_ or MOG_35–55_; 10 µg/ml final), recombinantly expressed and purified MOG_1–125_ (well coated with 10 µg protein) or of concanavalin A (2.5 µg/ml final) as positive control. T cell activation was evidenced by the incorporation of ^3^H-thymidine and measured in counts per minute (cpm). Shown here are the stimulation indices (cpm of peptide or protein-exposed cells over cpm of cells cultured in the presence of the irrelevant CNS antigen) +/− standard deviation. (TIFF 13,967 kb)

Supplementary material Fig. 6: Clinical scores of Lewis rats injected with AQP4_268–285_-specific T cells with and without NMO-IgG. On day 5 after T cell transfer, the animals of both groups showed partial loss of tail tonus (EAE score 0.5) as first clinical symptoms. In animals which received AQP4_268–285_-specific T cells without NMO-IgG, the clinical score was stable until the day of sacrifice (i.e. day 7), while the majority of animals which received AQP4_268–285_-specific T cells plus NMO-IgG progressed to a complete loss of tail tonus (EAE score 1). * p = 0.048 (Mann-Whitney U test). (TIFF 8845 kb)

Supplementary material Fig. 7: Characterization of astrocyte-destructive lesions in animals injected with AQP4_268–285_-specific T cells in the presence of NMO-IgG. Spinal cord sections of Lewis rats injected with AQP4_268–285_-specific T cells plus NMO-IgG (a-g) and with AQP4_268–285_-specific T cells plus control IgG (subcuvia, h-n) were reacted with antibodies against AQP4 (a, h, brown) or against GFAP (b,i, brown), with the ED1 antibody (c, j, brown), and with antibodies against C9 (d, k, red), rat IgG (e, l, brown), and CD3 (f, m, brown). Myelin was stained with luxol fast blue (g, n). Please note that the leakage of rat IgG was less pronounced when control IgG was used, most likely because there is no additional perivascular astrocyte damage. (TIFF 19,382 kb)

Supplementary material Fig. 8: Inflamed optic nerves of animals injected with AQP4_268–285_-specific T cells in the presence of NMO-IgG. Consecutive sections of the optic nerve were reacted with antibodies against AQP4 (a, brown) or against CD3 to identify T cells (b, brown), with the ED1 antibody to reveal activated microglia/macrophages (c, brown), and with antibodies against C9 (d, positive reaction products are red), rat IgG (e, brown) or human IgG (f, brown). Note that there was no evidence of AQP4 loss, and also no evidence of complement deposition, in spite of the presence of numerous T cells and activated microglia/macrophages. There was no leakage of rat IgG or human IgG into the optic nerve parenchyma. (TIFF 18,948 kb)
